# Experiences of Psychiatric Nurses Who Care for Patients with Physical and Psychological Violence: A Phenomenological Study

**DOI:** 10.3390/ijerph17145159

**Published:** 2020-07-17

**Authors:** In Ok Sim, Kyoung Min Ahn, Eun Jeong Hwang

**Affiliations:** 1 Red Cross College of Nursing, Chung-Ang University, Seoul 06974, Korea; hiraly@cau.ac.kr; 2 Saeyun Hospital, Chuncheon 24441, Korea; ahnkm0813@naver.com; 3 Department of Nursing, Sehan University, Yeongam-gun 58447, Korea

**Keywords:** experiences, psychiatric nurses, phenomenological study, physical and psychological violence

## Abstract

Introduction: The present study aims to understand the experiences and characteristics of nurses caring for patients with mental disorders characterized by aggressive behavior. Aim: The study aimed to understand and interpret the physical and psychological experiences and positive and negative aspects associated with nursing practices of patients with anger and aggressive behavior. Method: The participants of this study were twelve nurses with over three years of experience working in a mental hospital. More specifically, all our participants had experience caring for psychiatric patients with anger and aggressive behavior. The collected data were analyzed using the phenomenological analysis method and the procedure proposed by Colaizzi (1978). Result: The nurses’ experience was described in five categories: “fear of violence”, “exposure to a poor working environment”, “difficulty of emotional control”, “career regrets”, and “finding a solution to violence.” Discussion: The hospital should encourage and provide training sessions to teach nurses how to use proper intervention technique regarding medication and seclusion. Implications: The results of the present study suggest the need for ongoing hospital support and program development, intervention studies, and improvement of the work environment to resolve the burden of mental and physical difficulties experienced by psychiatric nurses.

## 1. Introduction

The lifetime prevalence of mental disorders in Korea, excluding nicotine use disorder, has increased to 23.1%, which subsequently increases better management procedures [1]. The national strategy of hospitalizing patients who exhibit aggressive and angry behavior has led to a surge in their numbers due to the social policy that allows the hospitalization of patients at a family member’s request [2]. Anger and aggressive attitudes in mentally ill patients not only cause problems in the community but also lead to complex issues in the family.

According to Iozzino, Ferrari, Large, Nielssen, and Girolamo [3], about 20% of patients admitted to an acute psychiatric ward exhibit violent behavior with unpredictable and angry offensive behavior. Anger and aggressive behaviors in mentally ill patients can be both sudden and unpredictable and can be directed at other patients and clinicians, thereby harming the health system environment [4]. Preventing acts of violence, such as anger and aggression, is essential; above all, prompt action from a properly trained nurse during the early stages of patient behavior can be a treatment turning point [5,6]. However, it is challenging to apply therapeutic nursing interventions in sudden, dangerous situations when patients behave aggressively. In addition, nurses have different perceptions of and attitudes toward nursing practice regarding the level of acceptance, judgement, and appropriate intervention for an individual patient’s behavior [7].

Nursing interventions for patients with a mental illness accompanied by anger and aggressive behavior include reporting to doctors, restraining the patient’s behavior, persuading the patient, isolating the patient, and administering oral medications as needed. There are times, however, when nurses are unable to mediate the insurmountable violence and aggressive behaviors, which may potentially lead to nurses observing violence without taking action [8]. In such situations, nurses in psychiatric wards can be the primary victims. Such violent circumstances cause stress, anxiety, and occupational burnout, leaving nurses with physical and mental trauma [9,10]. Between 25% and 80% of nurses working in acute care hospitals have reported experiencing patient violence in one form or another [11]. Many previous studies have reported that psychiatric ward nurses have low self-esteem and efficacy as compared to their peers working in general wards [12].

To date, preliminary studies of violence against psychiatric nurses include a meta-analysis of prevalence and risk factors [3,13], as well as an analysis of quantitative data targeting stressors in work performed by mental ward nurses [14]. However, there is a lack of research that provides an in-depth understanding and interpretation of psychiatric nurses’ experiences while caring for violent patients. Therefore, it is necessary to change enough social time and social awareness to prevent psychiatric nurses from being exposed to reality such as violence, to understand the causes of violence in psychiatric patients, and to better understand and prepare for preventive measures. In addition, further research is needed to recognize problems and change the environment based on experience in the nursing field.

Phenomenological research is a method by which a researcher understands the meaning of the experiences that each participant routinely experiences without prejudice. Rather than objective facts or knowledge of reality, this study is to understand the nature of how different individuals compose experiences, especially how to experience phenomena occurring in psychiatric wards, and to interpret principles of social phenomena [15]. The study aimed to understand and interpret the physical and psychological experiences and positive and negative aspects associated with nursing practices of patients with anger and aggressive behavior. Therefore, based on the results of this study, it is important to not only understand the nature of aggressive behavior of psychotic patients, but also to strengthen the positive elements of mental ward nursing and to help provide basic data for solving and preventing physical and psychological problems.

## 2. Materials and Methods

### 2.1. Study Design

In this study was used a qualitative methodology that employs a phenomenological approach to gain insight into the experiences of nurses currently working in psychiatric wards. The focus targeted their care for individuals with mental disorders characterized by anger and aggressiveness.

The study addressed the following overarching questions:

1. How do nurses describe their experiences related to violent patients with anger and aggressiveness in psychiatric hospital settings?
2. What are the nurses’ perceptions when dealing with aggressive and angry patients?
3. What has changed in the nurses’ professional/personal attitude and behavior as a result of these experiences?

### 2.2. Study Participants

A total of twelve nurses were selected as study participants. The reason for the final selection of 12 participants was the subject who was no longer referred to as new content as stated in the interview process. Specially, the participants of this study were those who had enough knowledge, experience and attitude to provide the most in-depth answers to research questions and were selected as those with more than 3 years of hospital experience. Particularly, the people who participated in this study were those working in the psychiatric ward of a general hospital in Korea, and were nurses with experience in caring for long-term patients who were hospitalized for up to 3 to 6 months under medical insurance.

The qualifications required for participants were as follows:

1. Over three years of work experience in a general hospital with over 300 beds.
2. Experience caring for acute patients with severe anger and aggressiveness in psychiatric ward.

All study participants signed written informed consent forms. The study was approved by the Ethics Committee (IRB: 1041078-201707-HRSB-144-01). The participants were informed that they could withdraw from the study at any time and that the collected data would be deleted afterwards.

### 2.3. Data Collection

The data were collected from October 2018 to November 2019. Participants in this research were experienced nurses who worked at psychiatric hospitals. The selected nurses were those who showed an interest and willingness to participate in this research.

The data collection was performed by recording and transcribing personal interviews based on semi-structured interview questions. The participants were informed that the collected data would not be used for any purposes other than those of the present study. We also explained the option of withdrawing from participation at any time and informed the participants that the collected information would be deleted on the day of termination of the research. After pre-structured prior consent, a semi-structured interview was made through recording, and the 1:1 interviews were conducted in a private meeting room and a small meeting room in the hospital, which allowed participants to feel comfortable. The interviews took at least 1–2 h, and additional individual interviews were conducted later if necessary.

### 2.4. Data Analysis

Using Colaizzi’s [15] phenomenological analysis method, the data were analyzed by the investigators. First, the story of the participants’ experiences was read as a whole to capture a sense of its significance. Second, after a text review, representative phrases in each participant’s statement were highlighted. Third, the highlighted comments were reconstructed into a general form. Fourth, the forms describing meaningful experiences were categorized by similar themes. Fifth, a cluster of themes was derived from the categories. Finally, the collected data were accurately summarized to represent the essential structure of the experiences.

### 2.5. Methods to Ensure Rigor

The trustworthiness of the findings was established using the criteria outlined by Guba and Lincoln [16], including credibility, transferability, dependability, and confirmability.

To ensure credibility and transferability, the results were presented for approval to the study participants. Additionally, the results of the study were presented to other nurses working in the same environment (i.e., not the study participants), and the results were confirmed by them. To ensure confirmability, we reviewed the relevant literature and tried to eliminate biases in the research process and the formulation of results.

## 3. Results

### 3.1. General Participant Information

The participants in this study were twelve nurses who currently worked in hospitals with over 300 beds. The participants’ information, including gender, age, workplace, and employment period, is shown in Table 1. While intervening with violent patients, the nurses faced physical and mental violence and were presented with occupation-related problems.

### 3.2. Classification of Psychiatric Nurses’ Experiences

The experiences of the psychiatric nurses were classified into five categories: “fear of violence”, “placed in a poor working environment”, “difficulty controlling emotion”, “become skeptical”, and “finding a solution to violence.” Table 2 shows twelve themes encompassing four categories and 210 meaningful participant statements. Further details are explained below.

#### 3.2.1. Category 1. Fear of Violence

Participants experienced unexpected physical violence when acute psychiatric patients showed violence. During violent incidents, study participants experienced difficulties in dealing with the situation and had suffered from repeated violent episodes.

Theme 1. Difficulty dealing with violence in the acute stage

Psychiatric nurses felt that it was best to wait for the patient’s violence to subside rather than to respond immediately due to safety concerns. The participants also had difficulty in coping with the discriminatory perceptions of some patients.

‘… Because safety is a priority, I tried to escape without considering the situation, and I asked the male staff for help’

(Participant 5)

‘… When violent patients refuse to talk, it’s best to wait until the violence subsides. It’s always best if no one gets hurt’

(Participant 7)

‘I don’t think there is much that a nurse can actually do in the acute phase. It’s also difficult for nurses to cope with a patient who tends to be biased towards women and less likely to listen’

(Participant 12)

Theme 2. Experiencing unexpected physical violence

Participants in this study pointed out that in the process of nursing a patient, they suddenly turned into a violent state and exerted physical violence. While using these forced restraints to stop these behaviors and protect the nurses themselves from danger, the nurses explained that they experienced beatings, kicking, biting, and even touching and hitting the entire body from the patient.

‘When they suddenly became aggressive, I notified the doctor, and then while injecting the medicine, the patient hit my face with his legs. Also, the patient spat on my face several times and threatened me using sexual insults or curse words’ (Participant 2).

‘… There was an alcoholic patient who couldn’t control his anger. One day, we were shocked by his outburst of anger; he threatened us with a knife’

(Participant 10)

Theme 3. Repeated violence hurting the soul

The study participants were found to have experienced heartache, which was more threatening than the situation, as they were repeatedly abused by a patient who was usually caring:

‘… even when I tried to understand patients, the violent situation that suddenly appeared made me feel betrayed and confused; It hurt me psychologically and spiritually and shocked me’

(Participant 3)

‘… at first, when the patient was violent, it was too much psychological pressure, and I experienced heartache’

(Participant 7)

#### 3.2.2. Category 2. Placed in a Poor Working Environment

The participants experienced difficulties in caring for patients due to the irrationality of the hospital structure and felt that the human rights of nurses were violated.

Theme 1. Experience with the irrationality of the hospital structure

The participants felt stressed about the heavy workload and resented the deterioration of their environment. The participants felt inconvenienced when listening to patients’ unreasonable demands. In this environment, the participants felt that it was not easy for them to cope with violence.

‘… I feel embarrassed and stressed out by a heavy workload and sudden outbreaks, and I am resentful of the environmental weakness’

(Participant 1)

‘… Unlike closed wards, open wards are weakly regulated. If the nurse refuses to listen to a patient’s demand, the hospital threatens to fire her. The patients take advantage of this hospital regulation’

(Participant 5)

‘… I think there is a limit to monitoring patients with insufficient manpower. It’s not easy to endure every aspect of a poor hospital environment where profit still has to be made despite the shortage of manpower’

(Participant 12)

Theme 2. Violation of human rights

When dealing with violence, the participants became passive in managing the situation because some actions may lead to legal issues. In the event of violence, medical staff members are less protected and seem to have more restrictions than patients.

‘… I am always careful of dealing with aggressive patients, because both the medical staff and the patient may be injured, and legal issues may occur. Even if I’m the only one injured, the hospital will not take action to sort things out. Despite the increasing amount of stress the staff members experience, the quality of welfare and the working environment do not seem to improve’

(Participant 2)

‘… Having reasonable judgment regarding a certain problem is important in any situation, but it doesn’t apply in real situations. In some situations, it is difficult for the medical staff to be understood, and I feel that the nurses have more restrictions than the patients. I think the nurses’ human rights are violated’

(Participant 4)

#### 3.2.3. Category 3. Difficulty Controlling Emotion

The participants experienced difficulties in controlling their emotions in violent situations. The nurses either kept their temper or, in the worst cases, ended up engaging in verbal and physical violence with patients. In such situations, the participants tried to stay calm.

Theme 1. Negative empathy with the patient

The participants were aware that violent situations should be handled in a calm and professional manner, but there were moments when they lost their temper.

‘The patient was screaming and threatening me using curse words. I forgot my role as a therapist at that moment, and I yelled back at the patient’

(Participant 1)

‘… When the patient insulted me, I continued to stay calm as if I was testing my own personal limits. By the time my emotions could no longer remain calm, I became violent as well when restraining the patient’

(Participant 4)

‘… Dealing with anger is inevitable. Sometimes I feel angry, become compulsive, and fight back’

(Participant 8)

Theme 2. Staying calm

Although the participants had trouble in controlling their emotions, they wanted to overcome their feelings by staying calm. As medical staff, they tried to focus on the therapeutic aspect.

‘… Although dealing with a violent situation imposes an emotional burden, I was able to stay calm by suppressing my anger’

(Participant 2)

‘… I try to stay as rational as possible and not be biased’

(Participant 4)

‘… I think that the violence occurs because they are psychiatric patients, and I try to control myself and see the therapeutic side only’

(Participant 8)

#### 3.2.4. Category 4. Career Regrets

The participants experienced negative emotions and regretted the fact that their career contained repeated violence. The participants wanted to change their jobs because they considered patients as persona non grata.

Theme 1. Skepticism about the job 

The participants experienced negative emotions and regretted having to deal with violence in their careers. They were also concerned about repeated violent incidents.

‘As the process of dealing with violence repeated, my self-esteem went down, and I lost my commitment and enthusiasm for this job. I think I need to get out of this job soon’

(Participant 1)

‘After experiencing violence from the patients, I was humiliated, ashamed, and depressed. I felt so regretful about my job that I even considered changing my career’

(Participant 2)

‘After losing my pride as a nurse, I felt a strong desire to do something other than being a nurse. My self-esteem went far down, and I was constantly feeling downhearted and depressed, which made me question my abilities. I also felt as if I was left alone and isolated from society, fearing no one would understand my feelings with sympathy’

(Participant 4)

‘When dealing with patients with anger and aggressive behavior, I am concerned that it [violent event] will repeatedly occur in other similar situations’

(Participant 8)

Theme 2. Feeling of avoiding the patient 

The participants regarded the patients expressing verbal violence with both fear and hatred and were reluctant to confront them while on duty. The patients’ lack of guilt and remorse for their violent behavior was also one of the reasons why the participants wanted to change their jobs.

‘Having confronted verbal violence on a daily basis, my perspective of a patient has changed from a person who inspires a desire to help them as a clinician to a person toward whom I feel fear and hatred, wishing to avoid them on my duty. Due to the frequent admission and discharge of these patients, it is hard to sincerely wish them well’

(Participant 1)

‘I had a patient who threatened to file a lawsuit against the hospital for overly aggressive restraint, and the hospital reluctantly compensated them with a settlement. Justifying his behavior, the patient bragged about the result to others, and I decided to leave my workplace and move to a different workplace in another region’

(Participant 5)

#### 3.2.5. Category 5. Finding a Solution to Violence

The participants had to constantly figure out ways to deal with violent behavior from patients. Having acknowledged the importance of drug treatment and restraint for violent patients in the acute stage, the participants proceeded to have a consultation with the patient in which the content was set on a patient-by-patient basis. They recognized that the patients’ violence was due to emotional factors stemming from their experience, and the patients felt remorse after the violent behavior, which deeply touched the participants’ hearts. The participants then attempted to establish a close rapport with the patients and tried to understand every aspect of the violence and the emotions expressed, which they were able to assess based on their years of clinical experience as they got older.

Theme 1. Importance of restraint and medical treatment

The participants considered medical treatment and restraint as critical methods for managing violent patients in the acute stage. They also mentioned that it is crucial to have a face-to-face conversation after the patient has eased his/her anger.

‘Restraint and medical treatment are essential clinical processes for patients to overcome further expressions of anger, and once this moment is over, we can then proceed to the next stage of having a conversation with the patient’

(Participant 11)

‘I’ve seen patients become calm after medication. After isolation, restraint, and drug injection in the acute stage of violence, it is important to have a consultation taking into considering the variation of the patient’s clinical condition’

(Participant 12)

Theme 2. Importance of building rapport

The participants recognized the patients’ violence was due to an emotional wound formed in the past, and that after the violent behavior, the patients often felt remorse. The participants also recognized the feasibility of nursing intervention for patients with whom they had a strong rapport.

‘We need to figure out ways to complement frequent admission and discharge of patients during the treatment process. It is best to cope with the patients by understanding the characteristics of psychiatric patients, which are verbal violence, temporary failure of emotional control, and immediate remorse after such violence, and forgiving them with generosity’

(Participant 1)

‘Isolation and medical treatment in psychiatric hospitalization may be effective in some cases. The most important process, however, is helping patients with sincerity through profound consultation and reducing their stresses or the pain they might have experienced in their family relationships’

(Participant 6)

‘At first, I feel threatened and hurt when the patients demonstrated violence, but as time passed, I soon got to feel a sense of compassion towards them’

(Participant 7)

‘When a patient failed to control their emotion and demonstrated aggressiveness, it was less challenging to provide nursing intervention to that patient, especially with those whom I had built rapport in advance’

(Participant 8)

Theme 3. The importance of working and years of experience

As psychiatric nurses, the participants highlighted the importance of several years of experience. The more clinical experience they had, the broader was their understanding of patients, and the greater their ability to stay calm and collected in various situations.

‘Aging, on the one hand, might be a disadvantage. Being older, on the other hand, allows me to understand and embrace any type of situation’

(Participant 3)

‘Having abundant lifetime experience itself is a major plus to work as a psychiatric nurse. I believe a clinician with extensive experience has a better understanding of the patient’s behavior’

(Participant 6)

‘Without much work experience, I had a hard time dealing with angry patients sometimes. As I gained further experience, I was able to stay calm, take control of my emotions, and managed to take care of the patients’ emotions’

(Participant 11)

## 4. Discussion

This study aimed to understand the experiences and characteristics of nurses caring for mentally ill patients who demonstrate aggressive behavior. For data analysis, the phenomenological analysis method and procedure proposed by Colaizzi [15] were used. The results showed that the experiences of nurses could be placed in five categories: “fear of violence”, “exposure to a poor working environment”, “difficulty with emotional control”, “feelings of betrayal and skepticism”, and “strengthening capacity to cope with violence.” Overall, twelve themes in four categories were identified in this study based on 210 meaningful statements.

The first category describes psychiatric nurses’ fear of violence. Although the nurses are constantly exposed to unexpected physical violence, the best way to handle the situation is to try to calm the patient, as repeated violent conditions can cause nurses to break down emotionally. According to a recent study, the overall rate of exposure to physical violence among nurses is 36.4% [17]. The rate is particularly high among psychiatric nurses who provide nursing care to psychotic patients. In a study conducted by Stevenson, Jack, O’Mara, and LeGris [11], nurses experienced not only unpredictable physical violence, such as being grabbed, kicked, spit at, or strangled, but also verbal violence. These results are consistent with our finding that our study participants reported feeling negative emotions, such as fear, shock, and numbness, during violent situations. These affected their ‘abilities to carry out their professional nursing roles’ [11]. Hence, creating a safe environment for both nurses and patients during violent events is a need that should be addressed by hospitals.

The second category, “exposure to a poor working environment”, suggests that the participants were stressed by overwork and the irrational structure of the hospitalization system; therefore, the nurses suffered from human rights’ infringement. Martinez [6] explained that nurses’ awareness of violence can vary, and that this variation is influenced by the working environment, particularly among those who work at emergency rooms or psychiatric wards. A heavy workload and a large number of patients are major problems in the nursing profession. Compared to other department nurses in psychiatric wards, where mutual interaction is considered crucial, failure to establish a nurse–patient relationship within a limited time adversely affects the general care process [18]. The hospital must, therefore, address manpower shortages to reduce workloads, increase efficiency, and provide better quality nursing care.

The third category, “difficulty with emotional control”, explains participants’ experiences with acting violently towards their patients. Our results align with those reported in a study conducted by Pazargardi, Fereidooni, Fallahi, Alijani, and Molazem [18], who found that when patients turn violent, nurses can also respond with aggressive behavior, which the patients find hard to accept; ‘this leads to an improper relationship between them’. In addition, the expression of nurses’ anger can be directed towards not only the patients but also towards their colleagues, such as other physicians and nurses. Such behavior negatively influences teamwork. Since violence is never acceptable, the participants must continue to maintain their composure. In addition, a quality working environment and mental health program must be created to improve the mental health of nurses and prevent them from becoming aggressive and expressing anger.

Concerning the fourth category, “having career regrets”, the participants experienced career regrets due to repeated exposure to violence, and they considered their patients as persona non grata, rather than nursing care recipients. This result is in line with the study conducted by Pazargadi et al. [18], who found that work exhaustion ‘diminishes their motivation to interact with the mentally ill patients’. The result also shares some similarities with the Study on a Psychiatric Nurse’s Mannerism Experiences [19], in which the nurses felt regretful for experiencing burnout and being numb to chronic mentally ill patients. From these responses, we can conclude that management of nurses’ mental health is necessary to prevent work exhaustion. It is also crucial for the nursing administration to address any problems or concerns and monitor staff job satisfaction to ‘encourage the retention of nurses at all stages of their careers’ [20].

In the fifth category, “finding solutions to violence”, the participants must continuously figure out how to deal with violent situations, and they mentioned that using isolation and medical treatment are key solutions for violent patients in the acute stage of anger. The participants also pointed out that they attempted to understand and establish rapport with the patients and having years of work and personal life experiences definitely helped them to cope with high-stress moments. According to Kaunomäki, Jokela, Kontio, Laiho, Sailas, and Lindberg [21], the most frequently nursing intervention for reduction in the risk of a patient’s violent behavior was pro-re-nata (PRN) medication and seclusion. PRN medication is used in the psychiatric ward for various reasons. Although it can decrease patient agitation, the study indicated that ‘there seems to be only limited evidence for its effectiveness’ [21]. Although the use of seclusion may be required to mitigate therapeutic reliance and the emergence of acute conditions, it can be seen as a negative intervention because ‘patients often consider the use of coercive methods as a form of punishment, ultimately aggravating the establishment of nurse-patient relationship’ [11,22]. The hospital should encourage and provide training sessions to teach nurses how to use the proper intervention regarding medication and seclusion. Another study conducted by Polacek, Allen, Damin-Moss, Schwartz, Sharp, Shattell, Souther, and Delaney [23] explained that nurses’ interactions with patients could improve their participation, which has a positive effect on the outcome and leads to patient satisfaction with care. Therefore, psychiatric nurses should create a healing culture of recovery-oriented treatment. Polacek et al. [23] also indicated that nurses’ patient care experiences enabled access to patient narratives in a sophisticated manner. Therefore, the hospital should not only create a treatment environment that facilitates nurse–patient interactions, but the experiences of novice nurses should be a basis for educating incoming nurses.

## 5. Conclusions

The present study using a phenomenological approach and analysis aimed to understand the experiences of psychiatric nurses caring for patients with serious emotional control issues. Based on the results of our data analysis, the nurses’ experiences can be classified into five categories encompassing twelve themes.

The study participants were fearful of violent situations, experienced difficulties in emotional control, and had career regrets. However, despite the ongoing violence, the nurses attempted to find ways to deal with the situation. The only solution seems to be improving the technical and emotional skills of nurses, as no intervention method can eradicate the emotional burden experienced by nurses, such as low self-esteem, career regrets, and losing pride and enthusiasm. The work environment, therefore, plays a crucial role in improving the quality of the nursing practice and ‘lowering psychiatric nurse reports of emotional exhaustion and depersonalization’ [24]. The importance of this study is to find solutions to their problems through the experiences of nurses in addition to previous studies. If there were limitations in the previous study that only resulted in positive and negative studies of nurses, this study provided basic data that could be used to recognize problems and consider solutions for each of the national, cultural and hospital systems in the future, which can be said to be meaningful. As a limitation of this study, due to the nature of qualitative research, we tried to exclude the prejudices or subjective judgments of researchers in the inquiry process, but the problem of the intervention of biased intention remains in the process of interpreting and understanding the subjective experience.

Based on the experiences and value of psychiatric nurses, further research is needed on program development and intervention studies that would improve the mental and physical difficulties currently experienced by the nurses. Moreover, regulations regarding general clinical safety must be reinforced to protect nurses from external and interpersonal issues, while maintaining an ethical stance and attention to patients’ legal rights.

## Figures and Tables

**Table 1:** General participant information.

Participant No.	Gender	Age	Work Place	Employment Period/Year	
				Hospital Career	Psychiatric Ward Career
1	Female	48	Hospital B	6	3
2	Female	35	Hospital Y	6	2
3	Male	46	Hospital A	20	15
4	Female	39	Hospital A	13	11
5	Female	60	Hospital B	32	7
6	Female	61	Hospital B	40	5
7	Female	63	Hospital S	10	10
8	Male	26	Hospital C	3	3
9	Female	60	Hospital S	10	3
10	Female	60	Hospital Y	8	6
11	Female	65	Hospital B	15	18
12	Female	58	Hospital T	25	4

**Table 2:** Classifications of categories and themes of psychiatric nurses’ experiences.

Category	Themes	Meaningful Statements
1. Fear of violence	Difficulty dealing with violence in the acute stage	Wanting to escape from violence due to safety concerns
		Waiting for the violence to subside
		Nurses’ actions in response to patients restricted
	Experiencing unexpected physical violence	The patient’s compulsive act of violence: hitting the nurse’s face with their legs
		Lacking the establishment of rapport / bitten or stoned by patients
		Threatened by an angry patient with a knife
	Repeated violence hurting the soul	Wounded feelings, shocked by the violence
		Feeling threatened when the patient becomes violent
2. Placed in a poor working environment	Experience with the irrationality of the hospital structure	Resentment of a poor environment due to heavy workload and stress
		Angered by patients claiming violation of their rights using hospital regulations that protect them
		A problematic work environment characterized by making a profit with low manpower
	Violation of human rights	The process of dealing with violence may raise legal issues
		Nurses feel more restricted than the patients
3. Difficulty controlling emotion	Negative empathy with the patient	Yelling back at the patient while forgetting role as a nurse
		Failure to control emotion, resulting in compulsive patient restraint and isolation
		Emotional damage is inevitable, feels the urge to fight back
	Staying calm	Experiences emotional burden, but suppresses feelings and maintains calm as a nurse
		Continues to stay professional and behave rationally
		Controlling emotions and recognizing the therapeutic aspects
4. Career Regrets	Skepticism about the job	Loss of self-esteem and a sense of duty
		Feeling depressed and hopeless
		Feeling disconnected and isolated from society
		Concern about recurrent situation
	Feeling of avoiding the patient	Feeling fear and hatred towards violent patients and considering them as avoid person
		Having career-regret moments when seeing the elated patient after an act of violence
5. Finding a solution to violence	Importance of restraint and medical treatment	At the moment of the patient’s violence, isolation is an essential treatment process considering the safety issue
		If violence occurs, patients should be isolated and given a medical injection. Consultations should be given afterwards considering the variations in patient clinical conditions
	Importance of building rapport	If a patient that shows violence has a well-established rapport with a nurse, they regret their behavior soon after
		Experienced changes in patients when trying to care for their wounded emotions through in-depth consultation
		Over time, nurses feel a sense of compassion towards the patient
		Feasible nursing intervention specifically for patients with well-established rapport
	The importance of years of experience	As the nurses become older, they can more easily understand that the patient’s violence is due to their previous experience
		Broad understanding of patients through years of experience
		As nurses gain more experience, they can stay calm and roll with the emotional changes of patients
